# Complete Genome Sequence of Salmonella enterica Bacteriophage PRF-SP1

**DOI:** 10.1128/MRA.00965-21

**Published:** 2021-11-24

**Authors:** Prasanna Mutusamy, Sasireigga Jaya Jothi, Su Yin Lee, Bent Petersen, Thomas Sicheritz-Ponten, Martha R. J. Clokie, Stella Loke, Andrew Millard, Sivachandran Parimannan, Heera Rajandas

**Affiliations:** a Centre of Excellence for Omics-Driven Computational Biodiscovery (COMBio), AIMST University, Kedah, Malaysia; b Department of Biotechnology, Faculty of Applied Sciences, AIMST University, Semeling, Kedah, Malaysia; c Section for Evolutionary Genomics, The GLOBE Institute, Faculty of Health and Medical Sciences, University of Copenhagen, Copenhagen, Denmark; d Department of Genetics and Genome Biology, University of Leicester, Leicester, United Kingdom; e Deakin Genomics Centre, School of Life and Environmental Sciences, Faculty of Science, Engineering and Built Environment, Deakin University, Victoria, Australia; Portland State University

## Abstract

We characterized the complete genome sequence of the lytic Salmonella enterica bacteriophage PRF-SP1, isolated from Penang National Park, a conserved rainforest in northern Malaysia. The novel phage species from the *Autographiviridae* family has a 39,966-bp double-stranded DNA (dsDNA) genome containing 49 protein-encoding genes and shares 90.96% similarity with Escherichia phage DY1.

## ANNOUNCEMENT

Members of the genus Salmonella are common foodborne bacteria worldwide that cause infections requiring antibiotic treatments (1). Lately, however, the emergence of multidrug-resistant (MDR) Salmonella strains has triggered interest in alternative treatments like phage therapy (2). To our knowledge, no prior reports on phage isolation from tropical rainforests are available. Considering the vast diversity of microenvironments they harbor, we anticipated the presence of diverse phages against bacterial pathogens. In line with this, we successfully isolated and sequenced a novel species of Salmonella phage, PRF-SP1, from a dry sandy soil sample (GPS coordinate, 5.4620°N, 100.1900°E) obtained 1 to 3 cm deep in a rainforest in Penang. The complete genome sequence of the phage is reported here.

**FIG 1:**
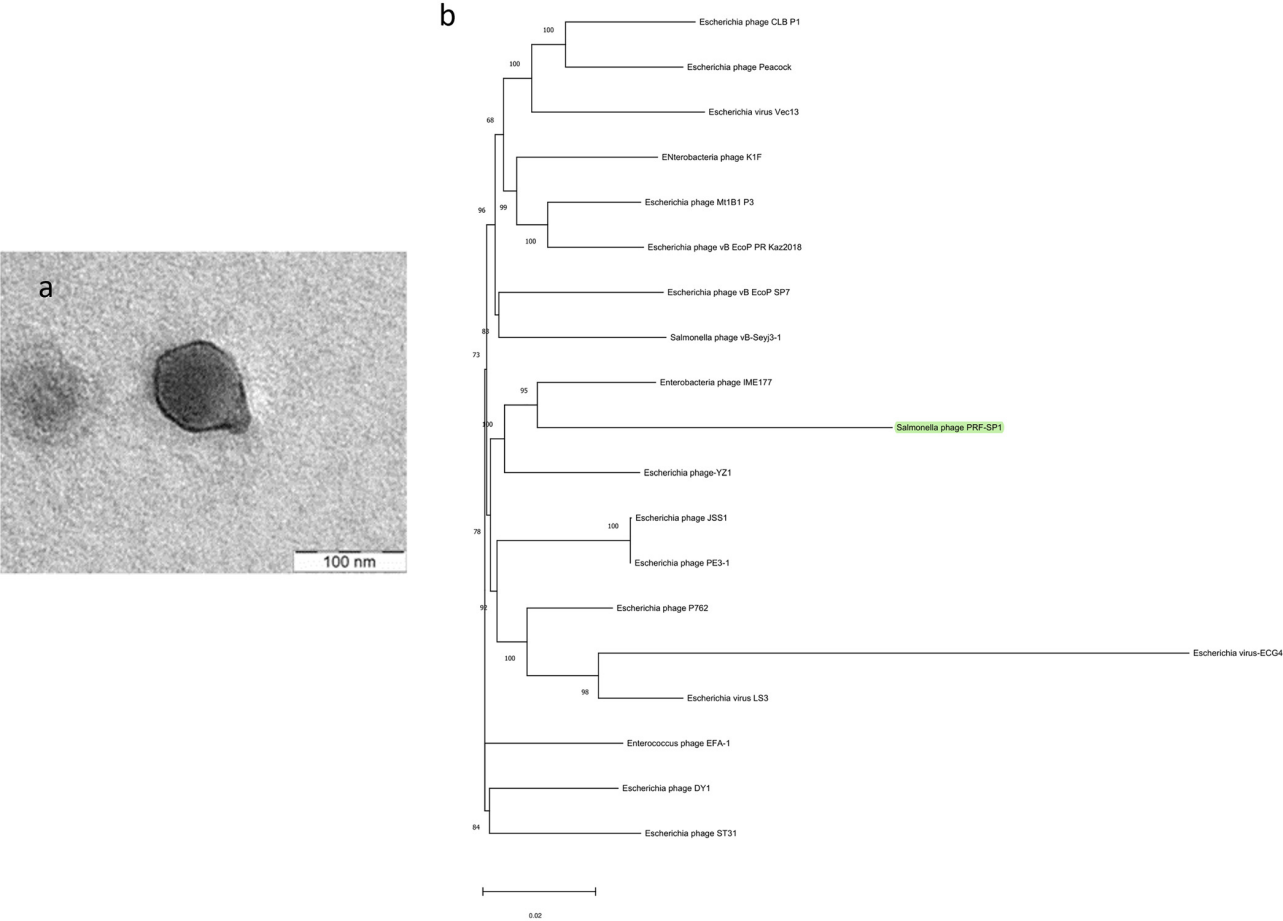
(a) Transmission electron microscopy image of phage PRF-SP1. The scale bar represents 100 nm. The size estimate of the virion was based on an average of 10 particles. (b) Phylogenetic analysis of Salmonella phage PRF-SP1. The top hits from BLASTn were used to identify related phages. The phylogeny was based on aligned core genes identified using Roary, with trees built using the neighbor-joining method with FastTree v2.1 (15). The bootstrap value was replicated 1,000 times (parameters, -boot 1000 -noml -nt), and the FastTree output was then visualized using MEGA v11.0.8. All phages identified were classified into the family *Autographiviridae*, subfamily *Studiervirinae*, and genus *Kayfunavirus*. Salmonella phage PRF-SP1 is highlighted in green.

Bacteriophage PRF-SP1 was isolated using the enrichment method (3) with Salmonella enterica Paratyphi A as its host, and it formed clear, circular plaques. The phage was then propagated to a high titer using the double overlay agar technique (4). The phage morphology was visualized using 1% (wt/vol) uranyl acetate and observed under a transmission electron microscope (TEM) at 40 kV (Fig. 1a). It possesses an isometric head (diameter, 58 ± 2.5 nm) and a cone-shaped tail stub (length, 23 ± 2.2 nm).

Phage DNA was extracted using phenol-chloroform (5) and quantified using a Qubit fluorometer. The DNA was subjected to a Nextera DNA Flex library preparation kit and sequenced using the Illumina MiSeq platform, yielding 83,303 reads with 300-bp paired-end sequences. The raw reads were assessed using FastQC v0.11.9 (6), before trimming using Trimmomatic v0.39 (SLIDINGWINDOW:4:28 HEADCROP:10 CROP:200 MINLEN:200) (7). Trimming resulted in 30,791 reads with a mean length of 200 bp. The genome was assembled using SPAdes v3.15.3 with default settings, before assessment using QUAST v5.0.2 (8). The final length of the assembled genome was 39,966 bp, with a GC content of 50.26%. Output from Bowtie2 v2.4.4 (9) revealed that a total of 97.36% of reads mapped back to the genome, with an average coverage of 308×. A PHACTS (10) analysis showed that PRF-SP1 is a lytic phage, while ResFinder v4.1 and VirulenceFinder v2.0 confirmed that no lysogenic factors or antibiotic resistance genes were found in the genome (11).

The assembled genome was annotated using Prokka v1.12 (12), which predicted the presence of 49 protein-coding genes; 23 had putative functions, and 26 were hypothetical proteins, with no tRNAs. Further analysis using BLASTn indicated that the phage had <95% average nucleotide identity with all other phages reported in the NCBI nucleotide (nt) database (top hit of ∼91% identity and 80% coverage with Escherichia phage DY1) and thus is representative of a new species based on the current standards (13). Further phylogenetic analysis was carried out with related phages which were identified by BLASTn through a fast core-gene alignment with MAFFT (used with -e and -n) using Roary v3.13.0 (14). As shown in Fig. 1b, the closest relatives of PRF-SP1 are *Enterobacteria* phage IME177 and Escherichia phage YZ1. All the identified related phages are part of the genus *Kayfunavirus.* The combination of genomic analysis, the presence of a gene encoding RNA polymerase, and phylogenetic analysis places PRF-SP1 as a new species in the family *Autographiviridae*, subfamily *Studiervirinae*, and genus *Kayfunavirus.* Based on this, we propose the new species “Kayfunavirus combio.”

### Data availability.

The complete genome sequence of phage PRF-SP1 has been deposited in the GenBank database under the accession number MZ923531. The associated BioProject, SRA, and BioSample accession numbers are PRJNA760259, SRR15809652, and SAMN21357400, respectively.
